# Methodological Framework for Multi-Cell Posturography Enabling Unconstrained Foot Placement and Open-Source Balance Assessment

**DOI:** 10.3390/s26196210

**Published:** 2026-09-30

**Authors:** Otto Hofstätter, Thomas Bochdansky, Anton Sabo, Mikael Bäckström

**Affiliations:** 1 Engineering, Mathematics and Science Education, Mid Sweden University, SE-831 25 Östersund, Sweden; mikael.backstrom@miun.se; 2 Life Science Engineering, University of Applied Sciences Technikum Wien, A-1200 Vienna, Austria

**Keywords:** multi-cell posturography, center of pressure, open-source, balance assessment, weight distribution index, biomechanics

## Abstract

**Highlights:**

**What are the main findings?**

- A multi-cell posturography framework using 16 distributed load cells across four mechanically decoupled sub-platforms successfully generates continuous Center of Pressure (COP) trajectories.
- The expanded hardware topography geometrically accommodates large anthropometric dimensions (up to EU shoe size 55) and natural foot rotation, while maintaining minimal mechanical crosstalk below 1%.

**What are the implications of the main findings?**

- The decentralized sub-platform design enables subjects to adopt a natural foot placement without requiring external optical tracking, reducing artifactual influences on postural control.
- Publishing the platform’s 3D-printable hardware blueprints and Python data pipeline as an open-source framework facilitates standardized, cost-effective balance assessments across different clinical and research facilities.

**Abstract:**

Background/Objectives: Computer-assisted posturography is utilized to quantify human postural control, yet existing dedicated systems frequently present physical constraints, such as limited sensing surfaces and rigid hardware barriers. In this study, a methodological framework is presented and a structural arrangement (OpenBalance) is designed to implement balance assessment accommodating a wider anthropometric range through zone-based foot placement using a decentralized load cell array, with preliminary implementation details provided in a repository. Methods: The configuration comprises an array of 16 discrete uniaxial vertical-force load cells embedded within four mechanically decoupled sub-platforms to minimize mechanical cross-talk. Biomechanical moment equations were implemented in a custom Python pipeline to compute a localized Center of Pressure (COP) for each sub-platform independently. A vector-based data fusion algorithm maps these local coordinates into a unified global coordinate system. The system evaluation incorporated baseline signal-to-noise ratio (SNR) analysis, mechanical crosstalk testing, and digital low-pass filtering. Results: A proof-of-concept evaluation using empirical data confirmed that the cascading coordinate model produces a continuous global COP trajectory and quadrant-specific load distributions. The platform dimensions (355 × 460 mm) and sensor topography geometrically accommodate natural external foot rotation (incorporating a 10° toe-out angle projection) and foot lengths corresponding to EU shoe sizes up to 55. Mechanical crosstalk between adjacent sub-platforms remained minimal (<1% of applied load). Conclusions: The OpenBalance framework confirms the technical feasibility of deriving a continuous global COP from a decentralized array of distributed load cells. While baseline component specifications and static verifications are established, comprehensive dynamic cross-validation against reference standards remains a necessary next step for the future development of open hardware that can be produced using 3D printing.

## 1. Introduction

A primary objective in computer-assisted dynamic posturography is to quantify human postural control with the highest possible level of detail. Consequently, various methodological approaches and instruments have been proposed and developed to assess posture in general and balance in particular. The market has seen numerous iterations, tracing back to the early recordings of Karl von Vierordt [1], who utilized a spring-loaded pen to transfer motion directly onto paper, up to contemporary, highly sophisticated, and room-filling installations such as multi-sensory virtual reality environments and biomechanics laboratories. This vast diversity of devices makes direct comparison difficult, even though various cross-methodological approaches exist—ranging from metrological instrument evaluations [2] and standardized mechanical sway simulations [3] to direct cross-device outcome comparisons [4] and mathematical reviews of posturographic parameters [5]. For the purpose of this work, measurement systems can be categorized into four distinct typologies: (1) non-dedicated posturography systems, such as general-purpose force plates and pressure-sensing mats, which are frequently utilized because they are readily available in research facilities [6,7,8]; (2) experimental setups designed for specific scientific studies and typically synthesized in review papers [9,10,11]; (3) dedicated stationary posturography systems [12,13]; and (4) dedicated mobile posturography systems [14,15].

**Figure 1:**
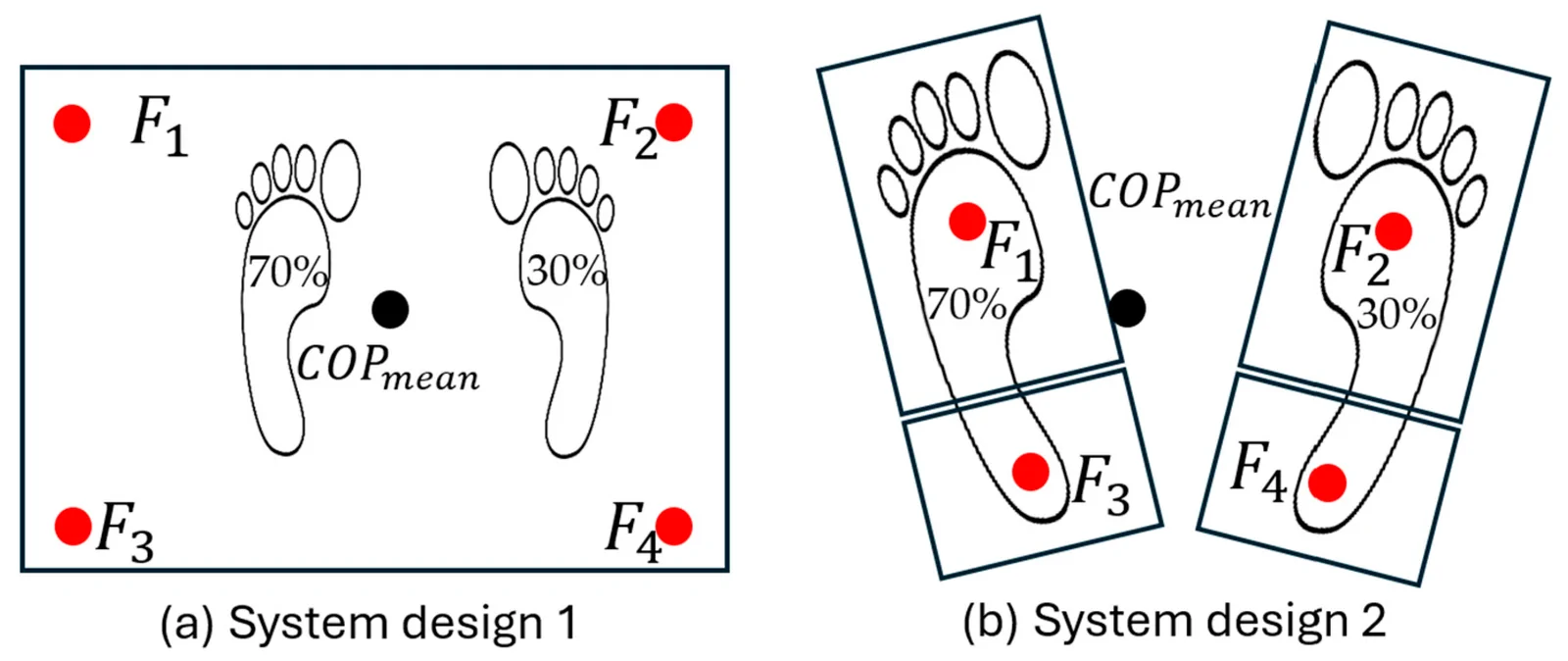
Mechanical differences between the two system designs of force plates (**a**) and posturography systems (Tetrax (Sunlight Medical Ltd., Ramat Gan, Israel)) with separate force plates (**b**). The tilted arrangement of the sub-platforms in the Tetrax system is designed to accommodate the natural average pronation of the feet. The red dots indicate the positions of the force sensors in the respective systems. The individually measured normal force components are labeled *F*_1_ to *F*_4_. The overall spatial metric is represented by the mean Center of Pressure over the duration of the measurement, labeled as *COP_mean_*.

Within the category of non-dedicated systems, conventional force plates offer excellent temporal and spatial resolution, enabling the precise detection of weight shifts. In contrast, pressure-sensing mats exhibit significantly lower temporal and spatial resolution per sensor element [2]. However, the prominent advantage of sensor mats lies in the flexible positioning of the subject on the sensing area. This self-selected foot placement prevents the subject from being forced into a specific posture. While general-purpose force plates also permit free placement, they suffer from the disadvantage that the absolute foot position on the plate cannot be determined without additional tracking. This exact relationship is illustrated in Figure 1. Even if a subject exhibits an asymmetrical weight distribution due to a postural deviation (e.g., 70% body weight on one foot and 30% on the other), a single monolithic force plate will only register the resulting combined center of pressure. Without external tracking, it cannot determine whether this shift stems from an altered foot placement or an uneven weight distribution between the feet.

Figure 1 contrasts the architectural and mechanical differences between a conventional monolithic force plate (a) and a multi-unit system with separate, mechanically decoupled sub-platforms (b). In a monolithic force plate (system design 1), the ground reaction forces are distributed across the fixed corners (*F*_1_ to *F*_4_) of a single continuous plate; while the global Center of Pressure and mean weight distribution can be computed, the exact spatial placement of the feet remains ambiguous without auxiliary tracking. Conversely, system design 2 utilizes decoupled sub-platforms (e.g., separate measurement platforms for each foot, which are further divided into heel and forefoot zones). When a subject steps onto specific sub-units—such as exclusively loading the heel sub-platforms—the normal force components are recorded locally by the dedicated sensors of that specific sub-plate. This physical decoupling ensures that regional loads are isolated directly at the respective sub-units, providing intrinsic positional insight that monolithic plates cannot resolve without external tracking. However, in conventional decoupled architectures utilizing a single central load cell per sub-platform (such as the Tetrax implementation), this approach does not resolve exact intra-plate foot placement. Rather, it must mathematically assume that the total normal force is applied exactly at a fixed, predefined spatial point—specifically, the geometric center of the respective zone. Furthermore, both conventional force plates and pressure-sensing mats present critical limitations during dynamic measurements, i.e., when the subject’s balance is actively or passively disturbed. Placing a heavy, conventional force plate onto unstable surfaces—such as balance boards—introduces significant inertial mass. This inertia creates an unnatural equilibrium state, distorting the biomechanical data acting like a mechanical low-pass filter. Conversely, when utilizing pressure-sensing mats during dynamic perturbations involving rapid jerks, shear forces adversely affect the sensor matrix.

Experimental posturography systems are prototypes and consequently suffer from a lack of commercial availability and standardization, rendering them inaccessible to external research facilities or clinical institutions. Dedicated stationary systems, on the other hand, exhibit highly heterogeneous features that complicate comparative analysis. Driven by commercial interests, these systems frequently induce vendor lock-in effects regarding both hardware and software, making standardized data exchange difficult or impossible. Furthermore, a major limitation is that both small-scale and large-scale systems are vulnerable to market discontinuation due to various commercial or strategic reasons [12,16]. Consequently, open-access approaches represent a highly promising pathway toward enabling the standardized comparison of measurement data across different systems [17].

Dedicated mobile systems exist in various configurations, ranging from wearable accelerometers and mobile pressure mats to portable force plates. Due to their highly specialized application fields, their metrological properties vary substantially. A prominent example is the Tetrax (Sunlight Medical Ltd., Ramat Gan, Israel) system (shown in Figure 1b System design 2), which features favorable characteristics for posturography due to its four independent force plates and provides a data interface for raw data export. However, because the system was originally designed for static assessments, its temporal resolution of 32 Hz is insufficient for capturing high-frequency dynamic perturbations [18].

To address these interconnected limitations, this study presents a posturographic framework that aims to combine the key advantages of different measurement approaches. The overarching purpose of this work is to provide a highly portable and cost-effective alternative to traditional posturography equipment, specifically designed for field assessments. In practical environments—such as athletic training facilities, sports associations, or out-of-lab clinical screenings—transporting and installing multiple monolithic standard force platforms is often logistically and financially unfeasible. By leveraging a decentralized open-source architecture, this approach seeks to overcome these practical barriers and expand access to mobile balance assessments for a wider audience.

To realize this goal, the primary technical objective of this manuscript is to establish the underlying mathematical relationships and design principles of the system. This theoretical foundation is supplemented by the practical implementation of a functional, 3D-printed hardware prototype. Furthermore, to support open-science practices and facilitate independent replication, the complete Python (version 3.14.5) source code for the evaluation pipeline, along with the mathematical framework and 3D-printable CAD files (STL format), are made openly available in a public GitHub repository: (https://github.com/dryride/OpenBalance).

## 2. Materials and Methods

### 2.1. System Design

The proposed methodology is based on a structural configuration utilizing an array of discrete force load cells. This approach aims to replicate and expand upon the four quadrant zones established by the conventional Tetrax system. However, these zones are designed with significantly larger dimensions to allow subjects to adopt a natural foot placement within the designated sensing zones during assessment. By enabling a flexible foot position within these zones, the artifactual influence of the measurement apparatus on the individual’s natural posture is minimized.

**Figure 2:**
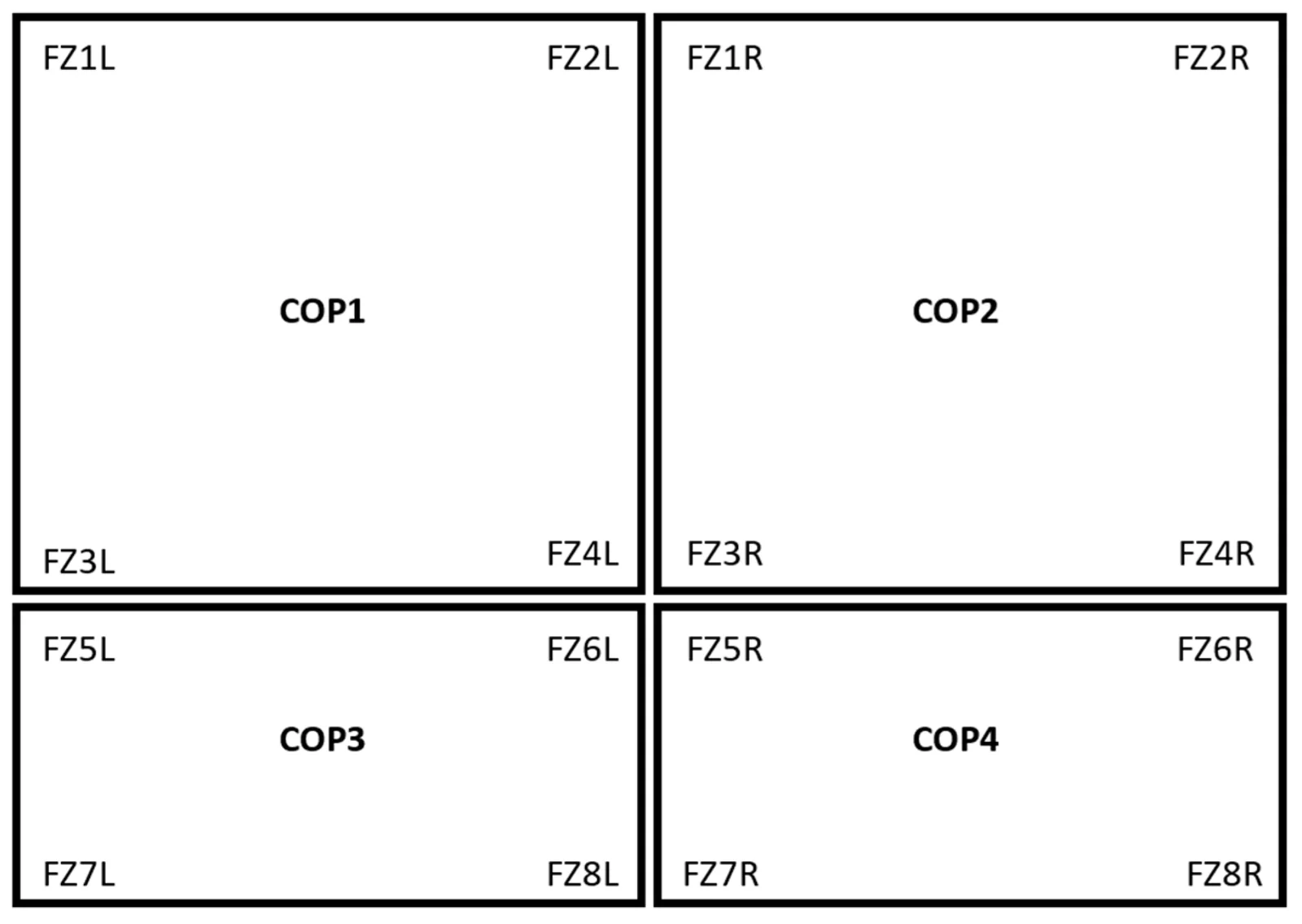
Schematic sensor topography and labeling convention of the distributed multi-cell array. Individual vertical force cells are labeled sequentially (FZ1 to FZ8) for the left (**L**) and right (**R**) sub-platforms using a row-major reading order. The sub-platforms feature a uniform width of 230 mm, with the forefoot and heel sections measuring 215 mm and 140 mm in length, respectively. The overall longitudinal dimensions were constrained by a shared, custom-built carbon testing shell.

Furthermore, the dimensional design of the new method (shown in Figure 2) explicitly addresses specific anthropometric constraints that limit conventional systems. Previous research has frequently attempted to assess human postural control using low-cost hardware components. However, in addition to constraints regarding temporal resolution, these systems often present severe limitations regarding their physical dimensions. For instance, on consumer-grade devices widely cited in the literature, such as the Wii Balance Board, subjects with a shoe size of approximately 45 EU or larger cannot place their feet properly within the intended sensing area. The framework presented here overcomes these physical restrictions by expanding the active measurement surface to accommodate a wider anthropometric range [9,19,20,21,22]. Specifically, the sub-platforms are designed with a uniform width of 230 mm, while the longitudinal dimensions differ between the anterior and posterior zones: the forefoot platforms have a length of 215 mm, whereas the heel platforms measure 140 mm in length. While the total system width of 46 cm (460 mm) significantly improves spatial capacity, the overall longitudinal dimensions were structurally constrained by an existing hand-laminated carbon shell (similar to a balance board). This shell was custom-built to be used interchangeably across multiple platform systems (e.g., Tetrax, Tymo (Tyromotion GmbH, Graz, Austria)) to ensure standardized testing conditions, thereby dictating the maximum length of the current hardware prototype.

**Figure 3:**
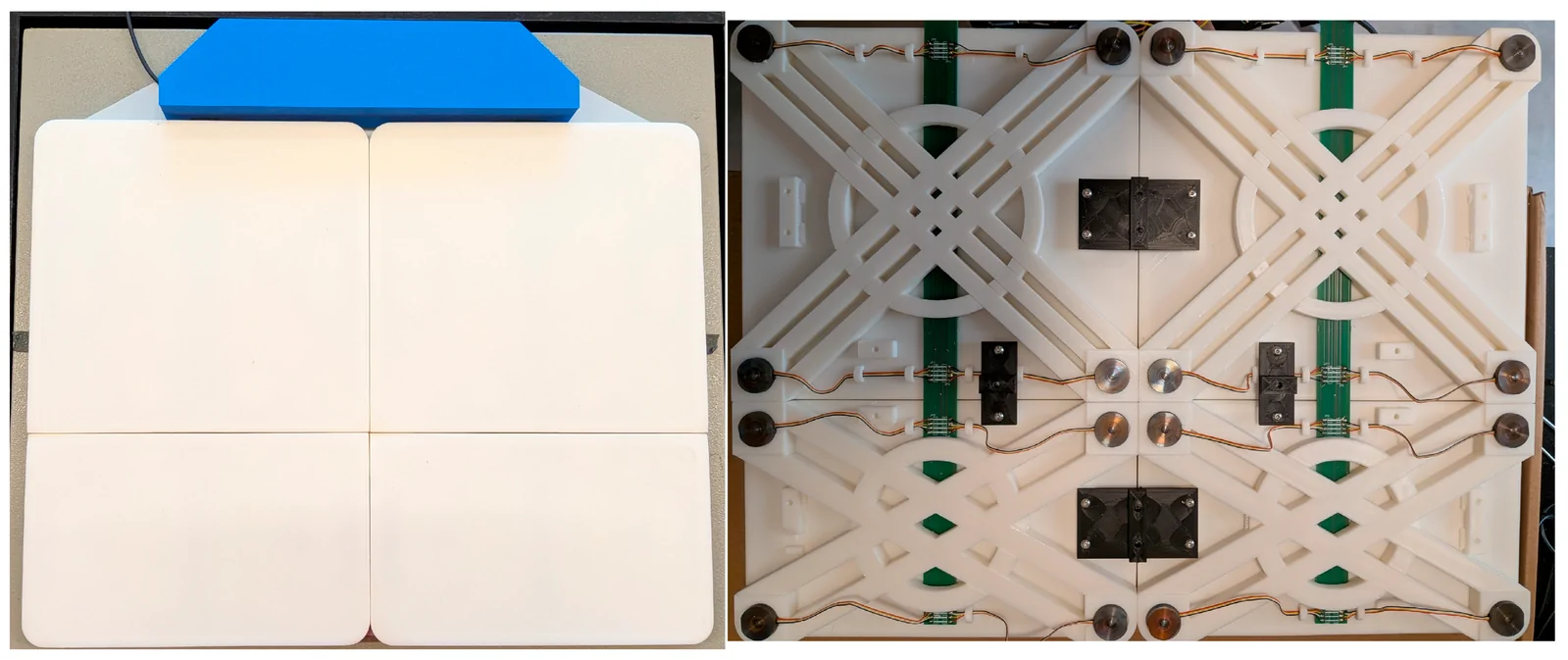
Hardware configuration of the developed platform prototype. The left image displays the top-view arrangement of the individual platforms (as described in Figure 2). The right image illustrates the bottom view, indicating the spatial distribution and location of the sensors. Note: In the depicted assembly state, only one of the two smaller connecting hinges between the anterior and posterior sub-platforms is mounted. The connection is split into two separate hinge plates to accommodate the central routing of the signal transmission board; the empty screw mounting points for the second hinge plate are visible adjacent to the mounted one.

Mechanically, the architecture operates as a system of four independent sub-platforms. These sub-platforms are mechanically decoupled and are intended to be interconnected exclusively via compliant mechanisms, such as living hinges or flexible joints, to prevent cross-talk and shear force distortions. To achieve high structural rigidity and direct regional force transmission, four discrete load cells are embedded directly within each individual sub-platform. A base plate featuring high surface hardness serves as the foundation, ensuring that forces from discrete contact points are accurately transmitted. Each foot rests on two sub-platforms of four load cells each, giving eight cells per foot and 16 cells in total. The exact arrangement and bottom-view layout are shown in Figure 3 in the Results section.

By utilizing four distinct load cells within each mechanically decoupled sub-platform, the system can calculate an independent local Center of Pressure (COP) for each of these specific units. This mathematical capability offers a significant advantage over conventional, monolithic force plates: it allows the system to determine the approximate spatial position of the respective heel or forefoot directly from the force data, without requiring external optical tracking. Furthermore, this architecture mathematically separates a subject’s local foot placement from their overall weight distribution. For instance, if a subject shifts more weight onto a specific sub-segment (e.g., the right heel) due to compensatory postures, the spatial coordinates and geometric assignment of that sub-platform remain fixed in the global frame. This weight shift mostly increases the magnitude of the vertical forces measured by those specific load cells, which in turn shifts the global COP calculation while keeping the underlying geometric transformation matrix constant. The following section presents the mathematical principles that enable this localized tracking and the subsequent global data fusion.

### 2.2. Data Analysis

#### 2.2.1. COP

In the first stage of the data processing pipeline, the local COP is computed independently for each of the four mechanically decoupled sub-platforms (i = 1, 2, 3, 4). Following established mechanical principles of moment equilibrium and lever arms [23], the local coordinates (*X_i_*_,*local*_;*Y_i_*_,*local*_) of the COP within each quadrant are derived from the vertical force components (*F_z_*_,*j*_) captured by the four discrete load cells embedded in each respective segment (shown in Equations (1) and (2)). The mathematical formulation for each sub-platform is defined as

(1) $$X_{i,\mathrm{local}}=\frac{\sum_{j=1}^{4}\left(F_{Z,j}*x_{j}\right)}{F_{i}}$$

(2) $$Y_{i,\mathrm{local}}=\frac{\sum_{j=1}^{4}\left(F_{Z,j}*y_{j}\right)}{F_{i}}$$

where *x_j_* and *y_j_* represent the fixed, known spatial coordinates of the individual load cells relative to the internal origin of the respective sub-platform, under the assumption that each rigid, decoupled sub-platform transmits a pure vertical reaction at each fixed cell coordinate, and the denominator represents the total vertical force (*F_i_* see Equation (3)) acting on that specific quadrant:

(3) $$F_{i}=\sum_{j=1}^{4}F_{Z,j}$$

To combine the localized sub-platform measurements into a unified posturographic trajectory, the local coordinates of each sub-platform must be mapped into a global coordinate system, regardless of the subject’s foot placement. This global coordinate transformation is achieved by incorporating the deterministic geometric offsets (*X_off_*_,*i*_;*Y_off_*_,*i*_), which define the physical center points of the independent sub-platforms relative to the absolute origin (0,0) of the overall system layout.

By applying a vector-based, element-wise data fusion, the global Center of Pressure (*X_global_*;*Y_global_*) is computed for each discrete time step as a force-weighted average of the transformed coordinates across all engaged sub-platforms (shown in Equations (4) and (5)):

(4) $$X_{\mathrm{global}}=\frac{\sum_{i=1}^{4}\left({(X}_{i,\mathrm{local}}+X_{\mathrm{off},i})*F_{i}\right)}{F_{\mathrm{total}}}$$

(5) $$Y_{\mathrm{global}}=\frac{\sum_{i=1}^{4}\left({(Y}_{i,\mathrm{local}}+Y_{\mathrm{off},i})*F_{i}\right)}{F_{\mathrm{total}}}$$

where *F_total_* (Equation (6)) denotes the absolute ground reaction force exerted across the entire measurement apparatus, which, under static situations, is the body weight:

(6) $$F_{\mathrm{total}}=\sum_{i=1}^{4}F_{i}$$

This cascading architecture naturally ensures spatial continuity and consistency when a foot spans across adjacent front and back sub-platforms, as each sub-unit’s local contribution is dynamically weighted by its respective vertical force (*F_i_*) relative to the total force (*F_total_*).

These mathematical formulations were subsequently implemented to develop a customized data analysis pipeline using the Python programming language (Python Software Foundation, https://www.python.org, accessed on 27 September 2026), utilizing the NumPy library for vectorized calculations. As detailed in the expanded derivation of Equation (7), substituting Equation (1) into Equation (4) and Equation (2) into Equation (5) yields an intermediate expression that explicitly highlights the individual contribution of each sub-platform. This expression algebraically simplifies into the final formulation, which reconstructs the direct force-weighted first moment of all 16 cells. Consequently, the two-stage cascading model is algebraically equivalent to a single global centroid and therefore introduces modularity without bias.

(7) $$X_{\mathrm{global}}=\frac{1}{F_{\mathrm{total}}}\sum_{i=1}^{4}F_{i}\cdot \left(\frac{\sum_{j=1}^{4}F_{i,j}\cdot x_{i,j}}{F_{i}}+X_{\mathrm{off},i}\right)\;\;\;\;=\frac{1}{F_{\mathrm{total}}}\sum_{i=1}^{4}\left(\sum_{j=1}^{4}\left(F_{i,j}\cdot x_{i,j}\right)+F_{i}\cdot X_{\mathrm{off},i}\right)\;\;\;\;=\frac{1}{F_{\mathrm{total}}}\sum_{i=1}^{4}\left(\sum_{j=1}^{4}\left(F_{i,j}\cdot x_{i,j}\right)+\sum_{j=1}^{4}\left(F_{i,j}\right)\cdot X_{\mathrm{off},i}\right)\;\;\;\;=\frac{1}{F_{\mathrm{total}}}\sum_{i=1}^{4}\sum_{j=1}^{4}F_{i,j}\cdot \left(x_{i,j}+X_{\mathrm{off},i}\right)$$

The resulting architectural capability is demonstrated in the Results section via graphical plots generated from empirical test data. Due to serial transmission overhead in this baseline proof-of-concept setup, the resulting sampling rate was nominal (~46 Hz) rather than strictly fixed. However, since the present methodological evaluation exclusively focuses on static, time-independent posturographic parameters (e.g., mean COP location and the Weight Distribution Index, WDI) rather than frequency- or velocity-dependent time-series metrics, this timing variation has no mathematical effect on the reported outcome measures.

#### 2.2.2. Weight Distribution Index (WDI)

To independently quantify static postural deformities, antalgic guarding mechanisms, or post-operative compensatory weight-bearing asymmetries, the static load distribution is often evaluated. For this purpose, the time-averaged vertical force of each decoupled quadrant is normalized against the total force to yield the percentage of weight-bearing per zone (*WD_i_* see Equation (8)):

(8) $$WD_{i}\left(\%\right)=\left(\frac{F_{i}}{F_{\mathrm{total}}}\right)*100$$

Following the clinical paradigm established by Kohen-Raz [18], the overall asymmetry of the weight distribution across the platforms is summarized via the Weight Distribution Index (WDI). Mathematically, the WDI quantifies the root-mean-square deviation of the four individual quadrant load proportions from the ideal physiological equilibrium of 25% per sub-platform, formulated as follows:

(9) $$\mathrm{WDI}=\sqrt{\frac{{\left(WD_{\mathrm{hl}}-25\right)}^{2}+{\left(WD_{\mathrm{hr}}-25\right)}^{2}+{\left(WD_{\mathrm{fl}}-25\right)}^{2}+{\left(WD_{\mathrm{fr}}-25\right)}^{2}}{4}}$$

where the subscripts denote the respective sub-platforms: heel left (hl), heel right (hr), forefoot left (fl), and forefoot right (fr). A WDI approaching zero indicates ideal bidirectional symmetry, whereas elevated values directly quantify the severity of the subject’s postural deviation (see Equation (9)).

#### 2.2.3. Hardware Configuration and Data Acquisition

To acquire the empirical test dataset, 16 discrete FX29 (TE Connectivity, Schaffhausen, Switzerland) force sensors were deployed in the physical topography described herein. The analog differential bridge signals from the load cells were digitized using two EVAL-AD7124-8-PMDZ evaluation boards (Analog Devices, Wilmington, MA, USA), each featuring a 24-bit sigma–delta analog-to-digital converter (ADC). The signals were amplified using the integrated Programmable Gain Amplifier (PGA) of the ADCs, configured with a hardware gain of 64. No external analog anti-aliasing filtering was used, leaving the input stage completely unattenuated. Hardware-level signal filtering was exclusively performed by the ADCs’ internal digital third-order sinc (sinc3) filter. Operating in Full Power Mode (fCLK = 614.4 kHz) with a register decimation factor of FS = 32, the ADCs achieved a maximum output data rate (ODR) of 6400 SPS. This specific digital configuration established an effective system bandwidth (−3 dB cut-off frequency) of 1740.8 Hz, with the first digital filter notch located at 6400 Hz.

The two ADCs were interfaced and addressed via a shared SPI (Serial Peripheral Interface) bus configuration. Signal aggregation and transmission were handled by an Arduino MKRZERO microcontroller unit (MCU), which streamed the digitized multi-channel data to a local personal computer via a standard serial interface (UART/USB) for persistent storage and subsequent analysis. Because timing was governed by the serial interface rather than a hardware-clocked sampler, the resulting sampling rate was nominal (~46 Hz) rather than fixed. A methodological limitation of the current baseline prototype is the absence of explicit hardware timestamps for individual data packets. Although the serial transmission overhead resulted in only minor temporal fluctuations, the lack of exact timestamps precludes post-hoc resampling (e.g., via piecewise cubic interpolation). Consequently, applying standard digital filtering or Fast Fourier Transforms (FFT)—which fundamentally assume equidistant time steps—to this specific dataset implicitly assumes a strictly constant sampling frequency. While this has no mathematical effect on the static, time-independent parameters evaluated in this study, future hardware iterations must incorporate explicit microsecond timestamping to enable precise resampling algorithms (e.g., Akima interpolation) for valid frequency-dependent processing.

#### 2.2.4. Testing Protocols and Signal Processing

An acquisition window of 30 s is widely established and validated in the posturographic literature [24,25]; consequently, this duration was selected for the baseline proof-of-concept trial. The measurement protocol requires the operator to ensure that the subject’s heels are positioned on the rear sub-platforms and the forefeet on the front sub-platforms. While positioning within each respective sensing zone accommodates minor natural variations in the base of support and foot angle, the feet are structurally constrained to these designated sub-platforms to ensure correct regional load isolation.

Prior to active data acquisition, the operator must perform a formalized initialization protocol across all 16 discrete load cells. Due to minor variances in the dead weight of the decoupled sub-platforms and sensor-specific drift, a baseline offset calibration is required. Specifically, a static zero-load measurement is executed for 30 s to extract the arithmetic mean of the vertical force signal under no-load conditions. Subtracting this baseline value element-wise from subsequent samples eliminates structural dead-weight artifacts and establishes a standardized, zero-referenced starting condition.

To evaluate the baseline noise floor of the decentralized sensor topography, the signal-to-noise ratio (SNR) for static loading conditions was calculated as *SNR* = 20log_10_(*μ*/*σ*), where *μ* represents the mean force and *σ* the standard deviation of the signal, in accordance with standard digital signal processing practices [26]. Subsequently, to mitigate environmental power frequency interference and ensure high-fidelity dynamic COP tracking, a digital low-pass filter with a cut-off frequency of 13 Hz was implemented [27]. Finally, to quantify potential mechanical cross-talk and verify the decoupling efficiency of the architecture, a standardized mass of 10 kg was sequentially placed onto each individual sub-platform while simultaneously recording the force outputs across all remaining unloaded sub-platforms.

## 3. Results

### 3.1. Baseline System Performance and Cross-Talk Validation

To evaluate the baseline noise floor, the acquired raw data was initially analyzed without digital post-processing. Under static loading conditions, the individual sensors exhibited an SNR ranging from 29.2 dB to 55.2 dB (filtered: 35.7 dB to 61.7 dB), with an average channel SNR of 47.0 ± 6.6 dB (filtered: 53.5 ± 6.8 dB). The high variance in baseline noise across individual channels can be attributed to unshielded wiring in the temporary breadboard setup. Due to the uncorrelated nature of the baseline noise, aggregating the 16 channels into the total vertical force yielded a singular systemic SNR of 54.8 dB (filtered: 61.6 dB).

Regarding the evaluation of mechanical cross-talk, the systematic loading tests confirmed the decoupling efficiency of the sub-platform architecture. When applying the static 10 kg load, the resulting cross-talk—evaluated as the root-mean-square (RMS) ratio of the steady-state parasitic force detected on adjacent sub-units relative to the applied load—remained minimal (below 1% of the loaded weight). This confirms that the mechanical separation and flexible joints effectively isolate the sub-platforms and prevent the undesired transmission of bending moments and shear forces.

### 3.2. Experimental Outcomes

As a result of the conceptual framework, a detailed sensor topography and coordinate system was established. The final system layout was realized using a total of 16 individual force cells arranged in a four-by-four matrix (distributed as two symmetrical two-by-four arrays for the left and right foot segments). The exact geometric arrangement and dimensioning of these individual force cells are shown in Figure 3 and are available in the repository.

**Figure 4:**
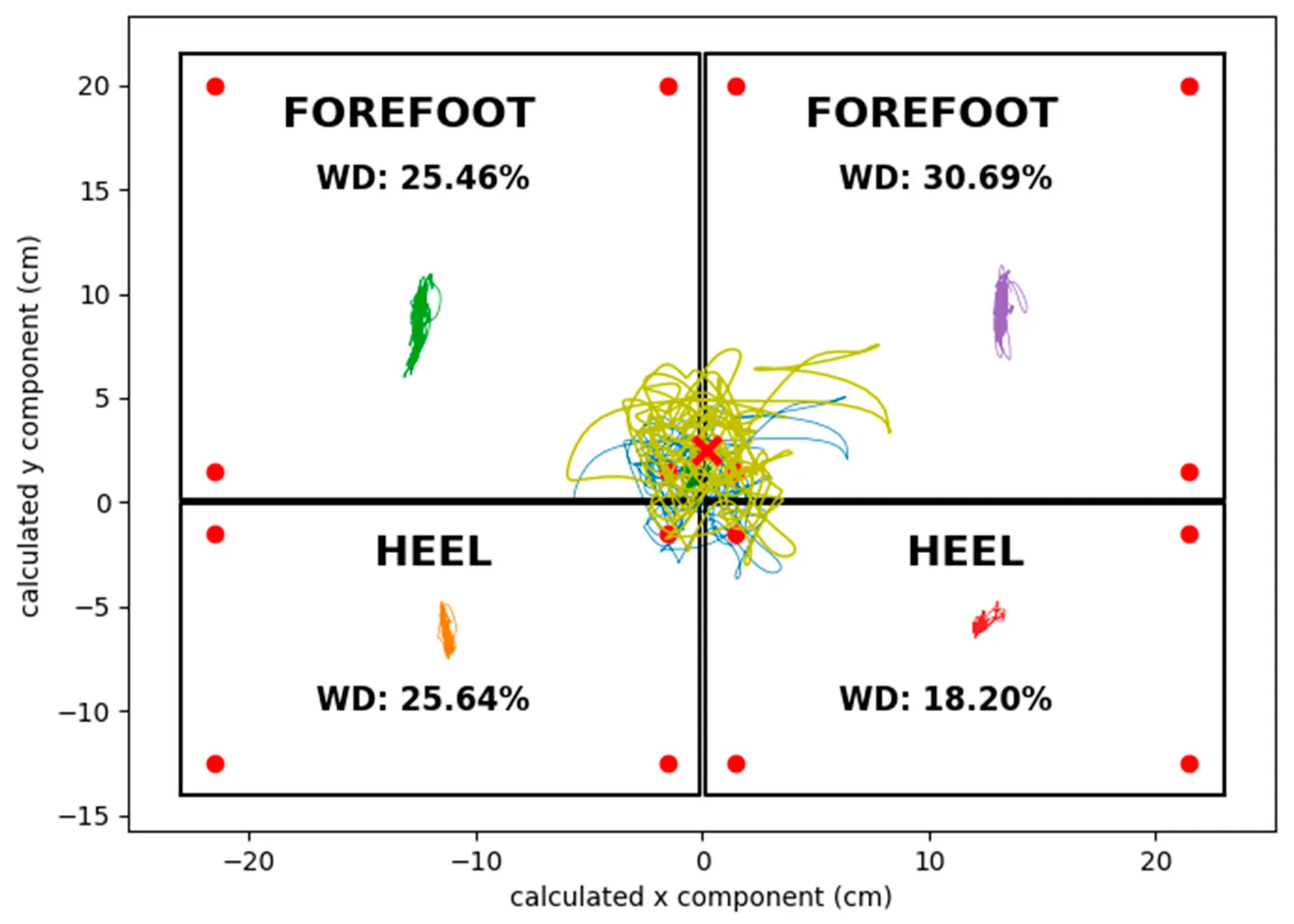
Graphical verification plot of the custom Python posturography pipeline demonstrating the cascading coordinate framework. Spatial dimensions on the X- and Y-axes are scaled in centimeters, representing the overall platform geometry (355 × 460 mm) optimized to accommodate natural external foot rotation. Red circles delineate the physical topography of the 16 integrated vertical force cells (FZ1 to FZ8 per side; see Figure 2). Furthermore, the colored paths represent the specific statokinesigrams (COP trajectories): green, purple, orange, and red indicate the local statokinesigrams of each respective segment. The blue trace represents the regional statokinesigram resulting from the summation of forces within that specific zone. The light green trace displays the newly calculated global statokinesigram of the entire system. Finally, the red ‘X’ denotes the mean position of all X and Y coordinate components.

Within this layout, each load cell is uniquely mapped within the coordinate system to facilitate precise sensor-to-channel assignment during data processing. The force cells are designated as “FZ” to denote the vertical force component, followed by sequential numbering from 1 to 8 for each respective side (left and right). The numbering convention follows a standard row-major reading schema. Accordingly, FZ8L represents the final sensor for the left foot. This scheme is presented in Figure 2 and built the basis for the results shown in Figure 4.

### 3.3. System Verification

As a primary result for the system verification, Figure 4 illustrates the graphical plot output generated by the custom Python data pipeline. The labels on the X- and Y-axes represent spatial dimensions scaled in centimeters. The overall physical dimensions of the platform assembly measure 355 by 460 mm. Incorporating the natural external rotation of the human foot, this expanded geometry geometrically accommodates foot lengths corresponding to EU shoe sizes up to 55 accommodating a natural, unconstrained foot angle within the designated sensing zones.

To ensure that the platform dimensions (355 mm length × 460 mm total width, providing a 230 mm width allocation per foot) comfortably accommodate extreme anthropometric dimensions—such as an EU shoe size of 55, corresponding to a maximum foot length of approximately 35.6 cm—a geometric projection analysis was applied. While a straight, unaligned foot of 35.6 cm would slightly exceed the 355 mm platform length, incorporating a natural external foot rotation angle (toe-out angle) of 10^∘^ yields a projected longitudinal length (*y*-direction) of 35.6 cm ⋅ cos(10^∘^) ≈ 35.06 cm. This geometric reduction ensures that the foot fits safely within the 355 mm boundary. Simultaneously, the transverse width allocation of 230 mm per foot provides ample clearance for the foot’s lateral width and its corresponding projection under rotation, preventing any physical constraint or edge-spilling.

In the graphical output, red circles are utilized to precisely delineate the physical positions of the integrated force cells. The individual measurement quadrants are labeled explicitly as “Forefoot” and “Heel” for both the left and right foot segments. Within each decoupled zone, the continuous trajectory of the localized sub-platform COP is plotted.

The center of the diagram displays two distinct posturographic trajectories representing the global COP tracked over the same 30-s duration. The background trajectory, rendered in blue, depicts the calculated global COP under the assumption that the localized center of force resides strictly at the geometric center of each sub-platform—effectively calculating the COP by merely summing the four load cells per quadrant. This computational approach directly replicates the evaluation methodology utilized by the proprietary Tetrax system. Layered over this baseline, the correctly transformed global COP is plotted in green, computed via the cascading coordinate framework and vector-based data fusion described in the methodology. Furthermore, the time-averaged center of these global coordinates is plotted as a red cross (“X”) directly over the trajectory.

The localized weight distribution (WD) is displayed as a percentage within each respective quadrant zone. The empirical test trial demonstrates a well-balanced bilateral load distribution between the left and right lower extremities. Specifically, the weight-bearing metrics capture 25.46% of the total load on the left forefoot, 25.64% on the left heel, 30.69% on the right forefoot, and 18.20% on the right heel. Due to standard decimal rounding for visual clarity within the graphical plot, the mathematical sum of these values yields 99.99%. Applying Equation (9) to these four quadrant loads yields a Weight Distribution Index of ≈4.45, quantifying the mild forefoot/heel asymmetry of the right limb in this trial.

**Figure 5:**
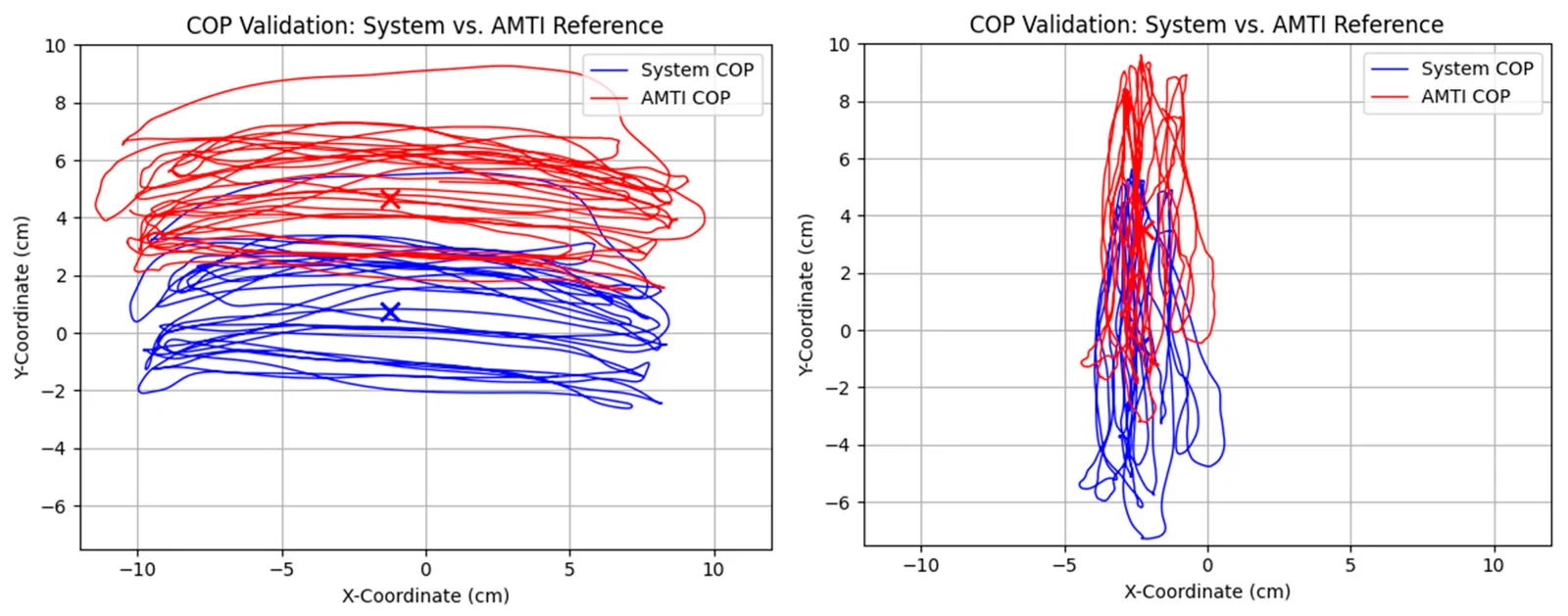
Validation of center of pressure (COP) tracking of the proposed system (blue line) against a reference force platform (AMTI—red line). (**Left**): Mediolateral weight shift showing the limits of stability (left-to-right). (**Right**): Anteroposterior weight shift showing the limits of stability (heel-to-toe).

To compare the spatial tracking of the OpenBalance framework with an established system, Figure 5 presents a qualitative visual comparison against a reference force platform (AMTI). The evaluation comprised two 30-s trials assessing the limits of stability (LOS): a mediolateral weight shift (left-to-right) and an anteroposterior weight shift (heel-to-toe). Because the reference platform and the proposed system utilize fundamentally different coordinate origins (corner vs. center intersection), the COP trajectory of the OpenBalance system was manually aligned to the AMTI reference frame. However, an intentional slight offset in the anteroposterior direction was maintained purely for visualization purposes. Without this vertical shift, the two trajectories would overlap heavily, obscuring the visual comparison of their morphology. As illustrated in Figure 5, the trajectory morphology of the proposed system (shown in blue) closely reflects the temporal movement pattern and directionality of the reference system (shown in red) across both axes. Visually, the maximum peak excursions at the extreme limits of stability appear slightly lower in the proposed system compared to the reference platform.

It should be noted that the current graphical output, as depicted in Figure 4, is intentionally minimalist, focusing strictly on the fundamental visualization of the COP trajectory and sensor topology for methodological validation. The development of a comprehensive graphical user interface—featuring detailed clinical legends, real-time metric textboxes, and automated reporting—is part of the ongoing evolution of the OpenBalance software pipeline and falls outside the scope of this initial proof-of-concept study.

### 3.4. Open Access Repository Architecture

Beyond the physical and mathematical validation, a key outcome of this research is the deployment of a fully open-access repository architecture, structured to ensure maximum scientific reproducibility and to serve as a centralized platform for future community-driven development. The repository design adheres strictly to standard software engineering conventions.

The computational core is housed within the /src directory, which contains the primary Python execution scripts responsible for the vectorized data processing and the cascading COP extraction. To allow external researchers to fully replicate and verify the findings of this study, the /data directory provides three independent raw empirical datasets. This includes the validation matrices “M2_left.txt” and “M3_right.txt”, which were utilized to confirm the sensor-to-channel assignments and local coordinate offsets for the left and right foot sub-platforms, respectively. Additionally, the file “M4.txt” contains the complete multi-channel data stream that generated the verification plot presented in Figure 4.

To bridge the gap between software execution and hardware implementation, the physical design constraints are shared within the /hardware_concept directory, which archives the engineering blueprints, primary dimensional constraints, and 3D-printable CAD files (STL format) of the platform assembly. Finally, the /docs directory is dedicated to archiving the peer-reviewed documentation and methodological preprints associated with this framework. The complete repository is persistently maintained and accessible to the public at https://github.com/dryride/OpenBalance (accessed on 27 September 2026).

## 4. Discussion

The empirical evaluation utilizing the OpenBalance proof-of-concept framework offers several critical insights regarding the physical assembly, signal acquisition, and future standardization of decentralized posturographic systems.

First, although the baseline verification trial supported the viability of the mathematical coordinate model, the analysis of the raw test data highlighted the sensitivity of decoupled multi-cell architectures to structural inconsistencies and manufacturing tolerances. While baseline zeroing is standard practice for conventional force plates, multi-platform systems require a formalized initialization protocol across all 16 discrete load cells to prevent local offset voltages from distorting the calculated weight distribution between independent zones. Due to minor variances in the dead weight of the decoupled sub-platforms and sensor-specific drift, the implementation of a 30-s baseline offset calibration protocol is strongly recommended prior to active testing. In future deployments, a static zero-load measurement should be executed across all 16 discrete force cells to extract the arithmetic mean of the vertical force signal under no load conditions. Subtracting this baseline value element-wise from subsequent samples will eliminate structural dead weight artifacts and establish a standardized, zero-referenced starting condition. This finding indicates that while raw setups provide highly promising preliminary results, such calibration routines are essential for cost-effective, decentralized architectures to achieve medical-grade initialization parity.

Second, regarding sensor dynamics, the deployment of distributed strain-gauge load cells is expected to mitigate the material-inherent hysteresis typical of flexible pressure-sensing mats. Standard conductive polymer mats suffer from viscoelasticity, transient creep, and phase distortions during dynamic load changes. In contrast, discrete strain-gauge load cells exhibit high structural rigidity. While full high-frequency dynamic characterization was outside the scope of this static proof-of-concept study—and will require the higher sampling rates proposed for future hardware iterations—the material properties of discrete load cells provide a more linear foundation for future dynamic extensions.

Third, while the current experimental setup achieved a sampling frequency of approximately 46 Hz—which was adequate strictly for evaluating static load distribution (WDI) and verifying the spatial coordinate fusion in this baseline proof-of-concept trial—the analysis confirms that the temporal resolution must be substantially increased for biomechanical applications. Although quiet standing frequencies typically reside below 5 Hz, reactive equilibrium corrections and ankle- or hip-stabilizing strategies in athletic or pathological cohorts elicit signals up to 15 Hz. Therefore, prescribing a baseline oversampling rate of 100 Hz or 200 Hz within the future OpenBalance architecture will successfully prevent spectral aliasing and signal distortion. This higher temporal resolution will ensure that raw posturographic data remains artifact-free and fully intact prior to any subsequent digital low-pass filtering [18,23,28,29].

While the AD7124 hardware supports sampling rates of up to 6400 SPS, the current system setup achieves an effective sampling rate of approximately 46 Hz. It is important to note that this is not a limitation of the ADC hardware, but rather of the effective sampling rate dictated by the current software implementation on the utilized microcontroller. Specifically, the processing overhead required to convert the 24-bit raw ADC data into double-precision floating-point values, combined with the transmission of these values as ASCII text via the serial interface, creates a significant communication bottleneck.

To achieve a target sampling rate of 100 Hz in future work, software optimizations will be implemented. By extracting the 24-bit raw integer data directly and replacing the ASCII transmission with a binary data stream, the microcontroller’s processing time can be drastically reduced. For target frequencies of 200 Hz and beyond, a microcontroller hardware upgrade is proposed. Utilizing a dual-core architecture (such as the RP2040 or ESP32) would enable the simultaneous, parallel polling of both ADCs. Alternatively, adopting high-performance microcontrollers with High-Speed USB capabilities—such as the Teensy architecture, which is widely recommended for high-speed data acquisition with this specific ADC library—would effectively eliminate the serial communication bottleneck entirely.

The empirical findings indicate that this temporal window yields a sufficient density of data points for baseline posturographic evaluation. Furthermore, the successful generation of the visual verification plot highlights the suitability of Python as a programming language for this framework. This suitability is primarily driven by its ecosystem of high-performance scientific libraries, such as NumPy (version 2.4.4) and SciPy (version 1.17.1), which enable vectorized, element-wise matrix manipulations. This computational efficiency allows Python to process multi-channel sensor streams and perform cascading coordinate transformations near real time, while libraries like Matplotlib (version 3.10.9) ensure publication-ready graphical representations of complex spatial trajectories.

A fundamental limitation of the current architectural framework is the utilization of uniaxial load cells, which only capture the vertical force component (*F_z_*). In true dynamometric systems, the COP calculation accounts for horizontal shear forces (*F_x_*, *F_y_*) and their resulting moments across the height of the top plate. However, extensive validation studies of structurally similar devices—such as the Nintendo Wii Balance Board—have demonstrated that during quiet, static standing, shear forces constitute a negligible fraction of the total force. Therefore, deriving the COP strictly from the vertical force distribution does not induce clinically significant errors in static scenarios. Nevertheless, future iterations aiming to incorporate high-impact dynamic perturbations may require thin-profile construction techniques to minimize the mechanical lever arm, or the integration of 3D force sensors to account for pronounced horizontal shear.

While the current baseline setup successfully validated the computational framework and coordinate fusion, transitioning this prototype into a fully deployable, open-source system requires specific verification milestones. First, while functional 3D-printable CAD blueprints (STL files) have been published in the repository to facilitate reproduction, future hardware revisions will aim to finalize a custom printed circuit board (PCB) to streamline microcontroller integration and minimize wiring overhead. Second, building upon the initial visual comparison against a reference system presented herein, rigorous quantitative dynamic cross-validation must be conducted. The proposed validation milestone involves mounting the fully assembled 3D-printed platform directly onto a laboratory gold-standard force plate. Standardized dynamic center-of-mass shifts will be applied by utilizing a mechanical testing device, such as a calibrated inverted pendulum or an automated load-shifting actuator. By quantifying the Center of Pressure (COP) trajectory deviations between the OpenBalance system and the reference plate under dynamic conditions, future studies can determine whether the platform’s signal quality and precision meet the thresholds required for clinical and athletic field applications.

## 5. Conclusions

To build upon the findings of this study, future research must embrace open-science paradigms, such as open-access source code sharing [17], to facilitate the collaborative implementation of posturographic algorithms. A central challenge in contemporary balance assessment is achieving cross-platform interoperability. Therefore, future initiatives will focus on developing a standardized, hardware-agnostic software framework capable of processing raw data from disparate proprietary systems—such as Tetrax setups or conventional monolithic force plates—using a single, unified software pipeline.

Another critical cornerstone for future validation is the optimization of the testing protocol. In proprietary systems like Tetrax, the standardized battery of positions is specifically tailored to stress and isolate vestibulocochlear and cervical proprioceptive pathways [18]. However, when evaluating healthy populations, the threshold at which measurable differences in structural perturbation or surface instability emerge remains an open debate. The empirical insights from other studies imply that healthy individuals can rarely be differentiated during simple, unconstrained static trials due to physiological ceiling effects [30].

To effectively differentiate athletic cohorts or track micro-progressions in high-performance sports, more demanding and dynamic perturbation protocols are mandatory. In our framework, such dynamic perturbations can be introduced mechanically by placing the decoupled platform system onto curved rocker elements (calottes), thereby inducing rotational surface instability while maintaining regional force tracking across the four sub-units. Consequently, the proposed software architecture must be expanded to support complex, multi-trial structures. Only by implementing structured sequences of multi-sensory challenges will it be possible to systematically isolate, compare, and quantify the discrete neuro-feedback loops—specifically the visual, somatosensory, and vestibular pathways—governing human postural control.

## Data Availability

The data are available in a GitHub repository, with the corresponding link becoming active upon publication. This repository provides the exact dimensions for constructing the platform, as well as a test program for the formulas detailed in the methodology section. By utilizing this program, researchers can input custom values to verify the underlying relationships of the COP formula. Link: https://github.com/dryride/OpenBalance.

## Abbreviations

The following abbreviations are used in this manuscript:

ADC	Analog-to-digital converter
CAD	Computer-Aided Design
COP	Center of Pressure
EU	European shoe size
Fx, Fy	Horizontal shear forces
Fz	Vertical force component
LOS	Limits of stability
MCU	Microcontroller unit
ODR	Output data rate
PCB	Printed circuit board
PGA	Programmable Gain Amplifier
RMS	Root-mean-square
SNR	Signal-to-noise ratio
SPI	Serial Peripheral Interface
STL	Stereolithography file format (3D printing)
UART/USB	Serial communication interfaces
WD	Weight distribution
WDI	Weight Distribution Index
